# MHD Flow of a Hybrid Nano-Fluid in a Triangular Enclosure with Zigzags and an Elliptic Obstacle

**DOI:** 10.3390/mi13020224

**Published:** 2022-01-29

**Authors:** Ines Chabani, Fateh Mebarek-Oudina, Abdel Aziz I. Ismail

**Affiliations:** 1 Department of Physics, Faculty of Sciences, University of 20 Août 1955-Skikda, 21000 Skikda, Algeria; fmoudina@gmail.com; 2 Mechanical Engineering Department, College of Engineering and Islamic Architecture, Umm Al-Qura University, Makkah P.O. Box 5555, Saudi Arabia; aiismail@uqu.edu.sa; 3 Mathematics Department, Faculty of Science, Tanta University, Tanta P.O. Box 31527, Egypt

**Keywords:** hybrid nano-fluid, triangular cavity, magnetic field, convective flow

## Abstract

The current study uses the multi-physics COMSOL software and the Darcy–Brinkman–Forchheimer model with a porosity of ε = 0.4 to conduct a numerical study on heat transfer by Cu-TiO_2_/EG hybrid nano-fluid inside a porous annulus between a zigzagged triangle and different cylinders and under the influence of an inclined magnetic field. The effect of numerous factors is detailed, including Rayleigh number (103 ≤ Ra ≤ 106), Hartmann number (0 ≤ Ha ≤ 100), volume percent of the nano-fluid (0.02 ≤ *ϕ* ≤ 0.08), and the rotating speed of the cylinder (−4000 ≤ w ≤ 4000). Except for the Hartmann number, which decelerates the flow rate, each of these parameters has a positive impact on the thermal transmission rate.

## 1. Introduction

Convection has always been the primary focus of research [1] for a variety of heating and cooling engineering systems. A modification of the geometrical shape of the system [2,3] was recently agreed upon among researchers in order to achieve spectacular optimization of the heat-transfer performance, from circular [4] and square geometries [5] to non-square geometries [6,7,8,9] (triangular, trapezoidal, etc.) as it has been proven by many research papers that aspect ratio and type of the geometry have a huge impact on thermal performance [10,11,12]. Heat-transfer fluid has also been the subject of significant development. Conventional fluids such as water and ethylene glycol now contain dispersed nanoparticles [13] that ensure an enhancement in thermal conductivity [14]. Therefore, these “nano-fluids” with improved physical properties yield great results, improving heat transfer [15]. Other attempts have been made [16,17,18,19,20] resulting in hybrid nano-fluids with two suspended nanoparticles, supporting the notion of lower costs and greateradvantages; therefore, they are regarded as enhancing parameters for heat systems, considering their superior thermal characteristics due to the combination of nanoparticles, when compared to mono nano-fluids and classical fluids [21,22].

In this context, Kahveci [23] studied heat transfer in a differentially heated square enclosure filled with nano-fluids and reported improvement in Nusselt number when inserting nanoparticles. Torki and Etesami [24] experimentally investigated the impact of the volume fraction of nano-fluids on Nusselt number in a rectangular enclosure and reported a proportional relationship between these two features.

Mansour et al. [25] explored an inclined square cavity with nano-fluids and heating circular solid, while Alsaberi et al. [26] inserted a hot solid square in a cold square under the effect of a magnetic field and reported a non-linear influence on heat transfer. Selcuk et al. [27] reported that increasing magnetic induction decreases velocity, which, in turn, decelerates the flow. The work of Zhang et al. [28] supports these results, finding a 33% reduction in cooling efficiency with increasing Hartmann number. Furthermore, Mahmoudi et al. [29] considered the Hartmann number parameter that restricted the heat-transfer rate. In this context, many studies were used to establish this research [30,31,32,33,34,35,36].

Ravnik et al. [37] studied natural convection in a cubic enclosure with a heated elliptical cylinder. Ishak et al. [38] numerically analyzed entropy generation in a classic trapezoid filled with alumina/water nano-fluid and an implemented immobile circular cylinder using the finite element method. Mebarek-Oudina et al. [39] studied the characteristics of a hybrid nano-fluid MHD flow in an annulus between a trapezoid and a rotating cylinder with a zigzagged wall.

Mahmoudi et al. [40] investigated nano-fluid flow in a triangular enclosure partially heated from below with a cold inclined wall. Majdi et al. [41] examined mixed convection in a lid-driven triangular enclosure with a motionless circle. Wang et al. [42] maintained a numerical approach for convection of a nano-fluid between a rotating circular cylinder and a conventional right-angled triangle, while Triveni and Panua [43] evaluated the impact of the aspect ratio of the hot wall in a triangular cavity and noticed a significant difference in the heat transfer rate as the caterpillar and zigzagged walls are more efficient than a linear wall. Other authors [44,45] studied convective flow inside enclosures with different hot-shaped inner cylinders. Several other references were utilized to establish this work [46,47,48,49,50,51].

These studies reported interesting results regarding the impact of motion, speed of rotation, placement of the internal cylinder, and the properties of nano-fluids, and it has been demonstrated that triangular-shaped enclosures are incredibly effective in the process of altering the efficiency of the heat system [52,53].

Based on the preceding investigations, this paper numerically analyzes thermal transport in a previously unseen configuration: an original triangular porous chamber filled with magnetized Cu-TiO_2_/EG hybrid nano-fluid and a spinning cylinder. The findings of this research will aid in achieving a better understanding of the effect of different cylinders inside the cavity, as well as expanding the contribution of sophisticated triangular geometries, which already have wide spread applications in industry [54,55,56] and are unquestionably adaptable in real-world applications, such as cooling electronic devices, solar heating systems, heat exchangers, solar collectors, etc. Aiming for high heat transmission efficiency under unusual settings, this work provides a significant contribution to future applications.

## 2. Physical Model

**Figure 1:**
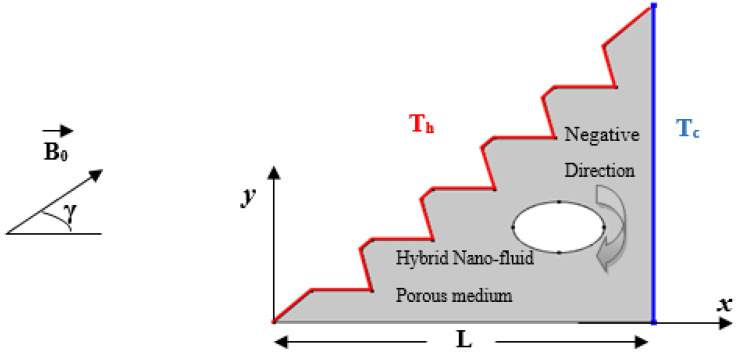
Configuration of the physical model.

This study provides numerical insight into convection, owing to the magneto-hydrodynamic flow of a hybrid nano-fluid in a porous triangular enclosure described by the Darcy–Forchheimer–Brinkman model. The configuration presents a right-angled triangular cavity of 1m length with a zigzagged inclined wall and an elliptical rotating cylinder along the x-direction inserted in the center of the geometry in order to investigate mixed convection; this is combined with heat transfer due to the buoyancy forces that lead tonatural convection and to the rotational velocity of the cylinder that drives forced convection. Figure 1 depicts the porous setup that is exposed to a magnetic induction, B_0_, detailed in Table 1, and filled with the Cu-TiO_2_/EG hybrid nano-fluid featured in Table 2. The right-angled wall is cold with a fixed temperature, T_c_, while the inclined wall is subject to a heating source and set as T_h_. The base of the triangle and the cylinder are both adiabatic.

Boundary conditions of the domain:

The right-angled wall: U = 0, V = 0 and T = T_c_; (1)

The inclined wall: U = 0, V = 0 and T = T_h_; (2)

(3) $$\mathrm{The}\;\mathrm{base}\;\mathrm{wall}:\;U=0,\;V=0\;\mathrm{and}\;\frac{\partial T}{\partial n}=0;$$

(4) $$\mathrm{The}\;\mathrm{cylinder}:\;U=w,\;V=0\;\mathrm{and}\;\frac{\partial T}{\partial n}=0.$$

## 3. Grid Test

**Figure 2:**
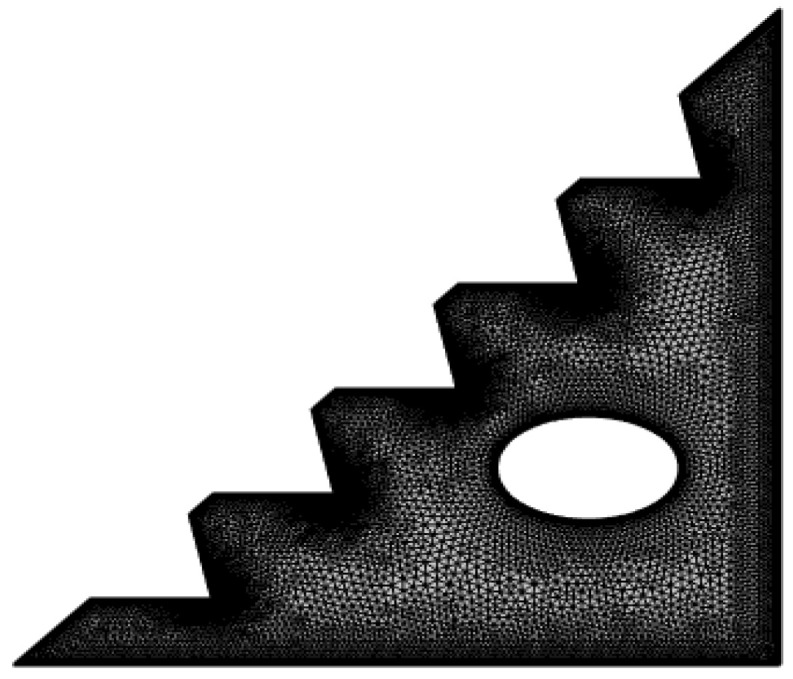
Mesh of the physical model.

The grid test is performed by studying several types of meshes in COMSOL multi-physics and assessing different parameters to enable the selection of the relevant mesh that provides valid results, considering our convective flow. Because algorithms in this software only provide high-quality elements with a value greater than 0.1 [57], our inquiry examined four types of elements indicated in Table 2 with satisfactory quality. The skewness measure quality is detailed; it allowed us to determine that increasing the element number improves the average quality of the mesh, indicating the dependability of the extra-fine mesh in numerical simulations. Furthermore, these four meshes performed an independence test at Ra = 10^5^ and *ϕ* = 0.04 to calculate the Nusselt number. Table 3 shows the obtained results, which reveal that the deviations in Nusselt number decreased as the mesh quality increased, and we can conclude that the mesh with the highest quality, “extra fine” ensured accurate outcomes. Therefore, the extra-fine mesh presented in Figure 2 was selected for our study.

## 4. Formulation of the Problem

### 4.1. Equations

The two-dimensional laminar convective flow in the studied triangular geometry is assumed to be stationary and incompressible. Thus, continuity and energy distribution in Cartesian coordinates are expressed as the following, according to [58,59]:

(5) $$\frac{\partial u}{\partial x}+\frac{\partial v}{\partial y}=0;\;$$

(6) $$\frac{\partial u}{\partial x}+\frac{\partial v}{\partial y}=0;\;$$

while the Navier–Stocks equations, which characterizethe flow of the hybrid nano-fluid, are described using the Darcy–Brinkman–Forchheimer model, which examines single-phase fluid circulation in a porous medium under the influence of magnetic fields [60], a model that has proven efficient and yields accurate outcomes [61]:

(7) $$\frac{1}{\varepsilon^{2}}\left(u\frac{\partial u}{\partial x}+v\frac{\partial u}{\partial y}\right)=-\frac{1}{\rho_{hnf}}\frac{\partial p}{\partial x}-\vartheta_{hnf}\frac{u}{K}+\;\frac{\sigma_{hnf}B_{0}^{2}}{\rho_{hnf}}\left(v\sin\left(\gamma \right)\cos\left(\gamma \right)-u\sin^{²}\left(\gamma \right)\right)-\frac{F_{C}}{\sqrt{K}}u\left|u\right|+\frac{\vartheta_{hnf}}{\varepsilon }\left(\frac{\partial^{²}u}{\partial x^{²}}+\frac{\partial^{²}u}{\partial y^{²}}\right);$$

(8) $$\frac{1}{\varepsilon^{2}}\left(u\frac{\partial v}{\partial x}+v\frac{\partial v}{\partial y}\right)=-\frac{1}{\rho_{hnf}}\frac{\partial p}{\partial y}-\vartheta_{hnf}\frac{v}{K}+\beta_{hnf}g\left(T-T_{avg}\right)+\;\frac{\sigma_{hnf}B_{0}^{2}}{\rho_{hnf}}\left(u\sin\left(\gamma \right)\cos\left(\gamma \right)-v\sin^{²}\left(\gamma \right)\right)-\frac{F_{C}}{\sqrt{K}}v\left|u\right|+\frac{\vartheta_{hnf}}{\varepsilon }\left(\frac{\partial^{²}v}{\partial x^{²}}+\frac{\partial^{²}v}{\partial y^{²}}\right);$$

where *K* is permeability, *Fc* is the Forchheimer coefficient, |*u*| is the amplitude velocity, and *T_avg_* is the average temperature:

$$K=\frac{\varepsilon^{3}d_{m}{}^{2}}{150{\left(1-\varepsilon \right)}^{2}},\;F_{c}=\frac{b}{\sqrt{a}\varepsilon^{\frac{3\;}{2}}}\;\mathrm{with}\;\left(a=150\;,\;b=1.75\right),\;\left|u\right|=\sqrt{u^{{}^{2}}+v^{{}^{2}}},\;T_{avg}=\frac{T_{h}+T_{c}}{2}$$

The following dimensionless numbers and variables are used to adjust the MHD flow distribution:

- Dimensionless numbers

$$Ra=\frac{\beta_{bf}g\left(T_{h}-T_{C}\right)L^{3}}{\alpha_{bf\;}\vartheta_{bf}}\;,\;Ha=LB_{0}\sqrt{\frac{\sigma_{bf}}{\mu_{bf}}}\;,Da=\frac{K}{L^{²}}\;,Pr=\frac{\vartheta_{bf}}{\alpha_{bf}}$$

- Dimensionless variables

$$\theta =\frac{T-T_{f}}{T_{h}-T_{f}}\;,Y=\frac{y}{L}\;,\;X=\frac{x}{L}\;,V=\frac{vL}{\alpha_{bf}}\;,U=\frac{uL}{\alpha_{bf}}\;,\;P=\frac{\left(p+\rho_{bf}g_{y}\right)L^{²}}{\alpha_{bf\;}{}^{²}\rho_{bf}}$$

The final governing dimensionless equations turn to:

(9) $$\frac{\partial U}{\partial X}+\frac{\partial V}{\partial Y}=0;$$

$$\frac{1}{\varepsilon^{2}}\frac{\rho_{hnf}}{\rho_{bf}}\left(U\frac{\partial U}{\partial X}+V\frac{\partial U}{\partial Y}\right)=-\frac{\partial P}{\partial X}-\frac{\vartheta_{hnf}}{\vartheta_{bf}}\frac{P_{r}}{D_{a}\sqrt{R_{a}}}U+$$

(10) $$\begin{array}{c} \frac{\sigma_{hnf}}{\rho_{hnf}}\frac{\rho_{bf}}{\rho_{hnf}}\frac{P_{r}}{\varepsilon }\frac{H_{a}{}^{²}}{\sqrt{R_{a}}}\left(V\sin\left(\gamma \right)\cos\left(\gamma \right)-U\sin^{²}\left(\gamma \right)\right)-\frac{F_{C}}{\sqrt{D_{a}}}U\left|U\right|\\ +\frac{1}{\varepsilon }\frac{\vartheta_{hnf}}{\vartheta_{bf}}\frac{P_{r}}{\sqrt{R_{a}}}\left(\frac{\partial^{²}U}{\partial X^{²}}+\frac{\partial^{²}U}{\partial Y^{²}}\right); \end{array}$$

(11) $$\frac{1}{\varepsilon^{2}}\frac{\rho_{hnf}}{\rho_{bf}}\left(U\frac{\partial V}{\partial X}+V\frac{\partial V}{\partial Y}\right)=-\frac{\partial P}{\partial Y}-\frac{\vartheta_{hnf}}{\vartheta_{bf}}\frac{P_{r}}{D_{a}\sqrt{R_{a}}}V+P_{r}\frac{\beta_{hnf}}{\beta_{bf}}g\theta +\;\frac{\sigma_{hnf}}{\rho_{hnf}}\frac{\rho_{bf}}{\rho_{hnf}}\frac{P_{r}}{\varepsilon }\frac{H_{a}{}^{²}}{\sqrt{R_{a}}}\left(U\sin\left(\gamma \right)\cos\left(\gamma \right)-V\sin^{²}\left(\gamma \right)\right)-\frac{F_{C}}{\sqrt{D_{a}}}V\left|U\right|\;+\frac{1}{\varepsilon }\frac{\vartheta_{hnf}}{\vartheta_{bf}}\frac{P_{r}}{\sqrt{R_{a}}}\left(\frac{\partial^{²}V}{\partial X^{²}}+\frac{\partial^{²}V}{\partial Y^{²}}\right);$$

(12) $$U\frac{\partial \theta }{\partial X}+V\frac{\partial \theta }{\partial Y}=\frac{\alpha_{hnf}}{\alpha_{bf}}\left(\frac{\partial^{2}\theta }{\partial X^{2}}+\frac{\partial^{2}\theta }{\partial Y^{2}}\right)$$

### 4.2. Validation

**Figure 3:**
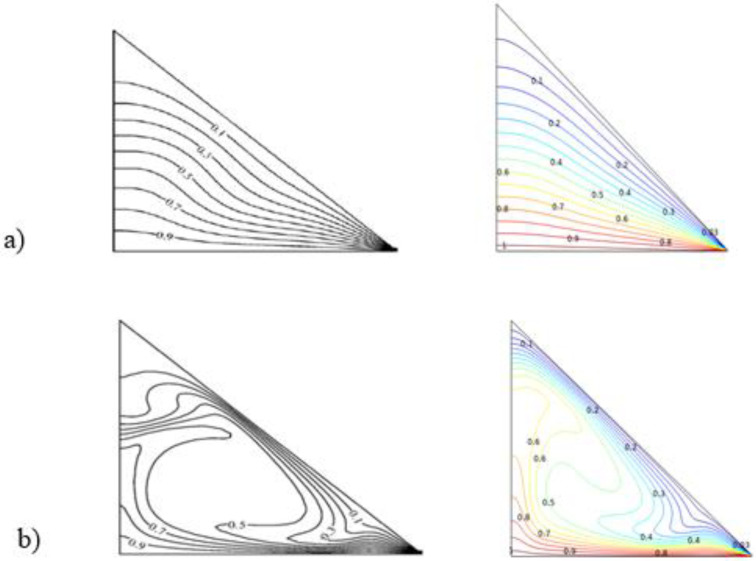
Isotherm and streamline comparison: previous work (left) and current work (right) for Ha = 0 (**a**), Ra = 10^4^ (**b**), and Ra = 10^6^.

The finite-element method (FEM) was used and applied in the COMSOL multi-physics software 5.6 to solve the dimensionless governing equations presented above with the given boundary conditions. The accuracy of the present program was assessed, and the results are reported in Figure 3, which compares the current isotherms and streamlines to those of the numerical findings of [62], in which natural convection was explored in a basic triangular enclosure.

### 4.3. Properties of the Hybrid Nano-Fluid

The volume fraction of the hybrid nano-fluid can be calculated as follows:

(13) $$\varphi =\varphi_{Cu}+\varphi_{{TiO}_{2}}$$

The equations of the specific heat capacity, density, thermal conductivity, thermal expansion, and electrical conductivity of Cu and TiO_2_ nanoparticles, respectively, were obtained from [63,64] and can be calculated as follows:

(14) $$Cp_{np}=\frac{\varphi_{Cu}Cp_{Cu}+\varphi_{{TiO}_{2}}\rho Cp_{{TiO}_{2}}}{\varphi }$$

(15) $$\rho_{np}=\frac{\varphi_{Cu}\rho_{Cu}+\varphi_{{TiO}_{2}}\rho_{{TiO}_{2}}}{\varphi }$$

(16) $$k_{np}=\frac{\varphi_{Cu}k_{Cu}+\varphi_{{TiO}_{2}}k_{{TiO}_{2}}}{\varphi }$$

(17) $$\;\;\;\beta_{np}=\frac{\varphi_{Cu}\beta_{Cu}+\varphi_{{TiO}_{2}}\beta_{{TiO}_{2}}}{\varphi }$$

(18) $$\;\;\;\;\sigma_{np}=\frac{\varphi_{Cu}\sigma_{Cu}+\varphi_{{TiO}_{2}}\sigma_{{TiO}_{2}}}{\varphi }$$

while the equations of the hybrid nano-fluid are as follows [63,64]:

The density of the hybrid nano-fluid is defined as:

(19) $$\rho_{hnf}=\left(1-\varphi \right)\rho_{bf}+\varphi \rho_{np}$$

where $\rho_{bf,}\sigma_{bf}$ are the density and electrical conductivity of the base fluid, respectively; and $\rho_{np},\sigma_{np}$ are the density and electrical conductivity of the used nanoparticles, respectively. Thus, the electrical conductivity of the hybrid nano-fluid is given by:

(20) $$\sigma_{hnf}=\left(1-\varphi \right)\sigma_{bf}+\varphi \sigma_{np}$$

As a result, the thermal expansion and specific heat of the hybrid nano-fluid are calculated to as follows:

(21) $${\left(\rho \beta \right)}_{hnf}=\left(1-\varphi \right){\left(\rho \beta \right)}_{bf}+\varphi {\left(\rho \beta \right)}_{np}$$

(22) $${\left(\rho Cp\right)}_{hnf}=\left(1-\varphi \right){\left(\rho Cp\right)}_{bf}+\varphi {\left(\rho Cp\right)}_{np}$$

while the thermal and electrical conductivity of the hybrid nano-fluid are obtained from the following equations:

(23) $$\frac{k_{hnf}}{k_{bf}}=\frac{k_{np}+\left(n-1\right)k_{bf}-\left(n-1\right)\left(k_{bf}-k_{np}\right)\varphi }{k_{np}+\left(n-1\right)k_{bf}-\left(k_{bf}-k_{np}\right)\varphi }$$

(24) $$\frac{\sigma_{hnf}}{\sigma_{bf}}=1+\frac{3\left(\sigma_{np}-\sigma_{bf}\right)\varphi }{\left(\sigma_{np}+2\sigma_{bf}\right)-\left(\sigma_{np}-\sigma_{bf}\right)\varphi }$$

Thermal diffusivity of the hybrid nano-fluid is considered as:

(25) $$\alpha_{hnf}=\frac{k_{hnf}}{{\left(\rho Cp\right)}_{hnf}}$$

The dynamic viscosity is given as follows, according to the Brinkman model [58]:

(26) $$\mu_{hnf}=\frac{\mu_{bf}}{{\left(1-\varphi \right)}^{2.5}}$$

The thermo-physical characteristics of the hybrid nano-fluid used in this study are presented in Table 4 [65].

## 5. Results and Discussion

This section will provide the numerical results obtained by streamline and isotherm contours, as well as the average Nusselt number for three major parameters: Rayleigh number (10^3^ ≤ Ra ≤ 10^6^), to study the convective heat transfer in the laminar regime and explore its features near the transition mode; Hartmann number (0 ≤ Ha ≤ 100), in order to investigate the relation between magnetic-field strength heat-transfer efficiency; and the volume fraction of the hybrid nano-fluid (0.02 ≤ *ϕ* ≤ 0.08), to evaluate the presence of nanoparticle in a porous medium with constant properties: Darcy number, Da = 0.1; porosity, ε=0.4. Additionally the following geometrical factors are discussed: the impact of the rotation of the internal cylinder with a speed, w, of (−4000 ≤ w ≤ 4000), as well as the placement of the cylinder and several shaped obstacles (square, circle, elliptic, and triangle).

### 5.1. Impact of the Nano-Fluid Volume Fraction

**Figure 4:**
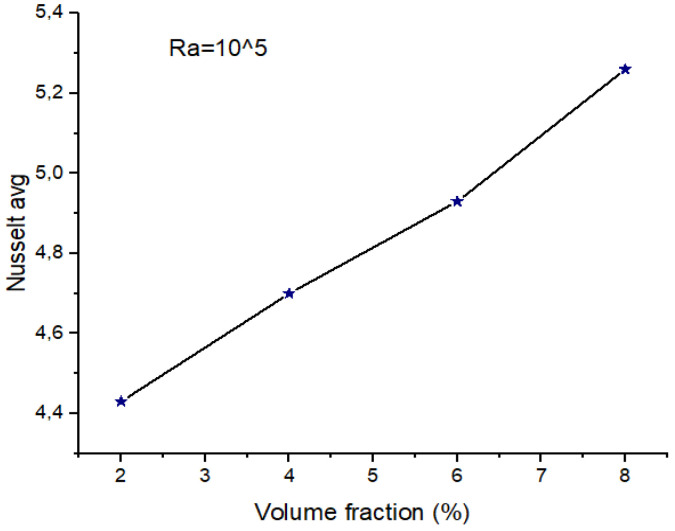
Average Nu for Ra = 10^5^, Ha = 0, and w = 0.

At Ra = $10^{5}$(Figure 4), the average Nu number appears to grow with the volume fraction where natural convection significantly dominates. Augmenting the concentration of the hybrid nano-fluid corresponds to an increase in the presence of nanoparticles, both Cu and TiO_2_, that present enhanced thermo-physical characteristics compared to classical fluids, as presented in Table 4, particularly their thermal conductivity. It worth mentioning that these properties improve the thermal conductivity of the hybrid nano-fluid and also increment the surface area of the nanoparticles [58]. Therefore, Nu_avg_ is proportional to the presence and the volume fraction of the hybrid nano-fluid, and such correlation contributes to convective transfer.

**Figure 5:**
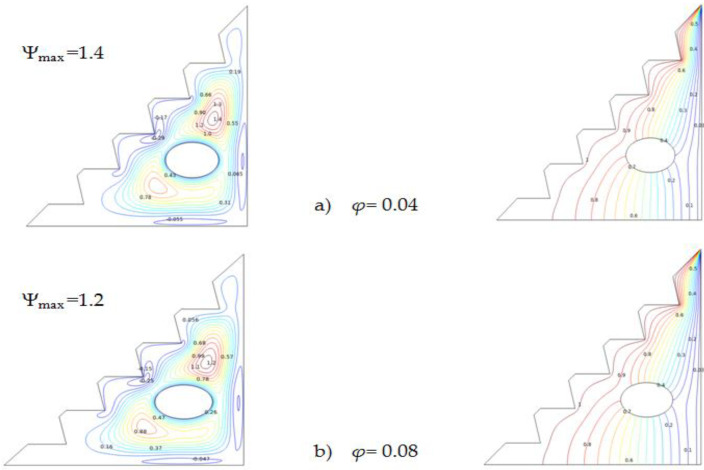
Streamlines and isotherms for Ra = 10^5^, Ha = 0, and w = 0.

Figure 5 presents the impact of the hybrid nano-fluid concentration on heat-transfer features, isotherms, and streamlines. It is shown that incrementing the volume fraction of the nanoparticles has only a minor effect on streamline and isotherm contours, with a slight modification to the detected stream-function value. The enhanced heat transfer properties provided by the increase in the volume fraction also intensify entropy generation [66,67], which results in decremented stream-function values and a reduced heat-transmission rate.

Although the presence of nano-fluids is intended to boost convection, increasing their concentration also increases entropy production. Therefore, an equilibrium must be considered when augmenting *ϕ*.

### 5.2. Impact of Rayleigh Number

**Figure 6:**
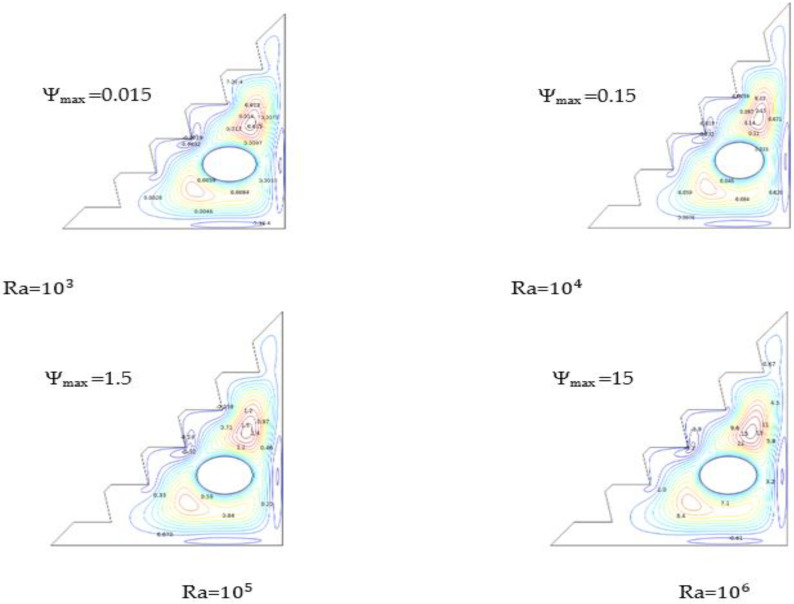
Streamlines for Ha = 0, w = 0, and *ϕ* = 0.02.

Figure 6 illustrates the influence of Ra number on streamlines for 2% of the hybrid Nano-fluid and no presence of the magnetic field. The results reveal that increasing Ra causes linear growth in the stream functions.

It is shown in Figure 6 that an increasing Ra number causes the vortices in the surroundings of the cylinder to grow larger and stronger, enabling the free convection to be significantly exploited, which strengthens the velocity field and speeds up the flow. As a result, the hybrid nano-fluid is heated up and becomes less dense, allowing buoyancy forces to ascend the hybrid nano-fluid and focus the flow on the upper part of the cylinder. Therefore, heat transfer is a trend of Rayleigh number since its enhancement indicates better heat transmission.

This enhancement is noted to be in a high range for Ra = $10^{6}$, with a stream-function value a hundred times greater than Ψ_max_ at Ra = $10^{4}$ and ten times greater than Ψ_max_ at Ra = $10^{5}.$ These results show that approaching the critical Rayleigh value (almost $10^{9}$) engenders significant values of heat transfer.

### 5.3. Impact of Hartmann Number

**Figure 7:**
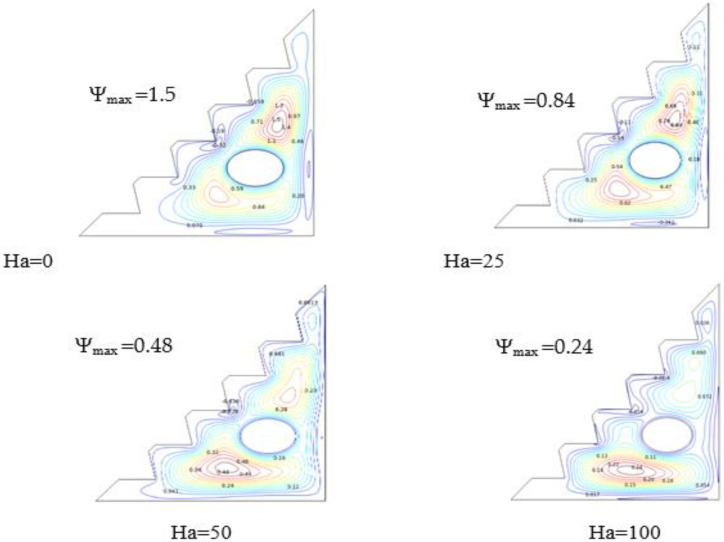
Streamlines for Ra = 10^5^, w = 0, and *ϕ* = 0.02.

On the other hand, as shown in Figure 7, the presence of a magnetic field diminishes the convective transfer rate, as Lorentz forces inhibit the development of vortices and thus reduce the velocity of the nano-fluid and suppress the flow distribution. In the case where no magnetic induction was introduced, two large vortices were formed around the cylinder, which smoothed the flow of the hybrid nano-fluid. With an increasing Hartmann number, stream-function values were reduced as vortices shrunk in size and nearly disappeared in the upper part of the enclosure, causing the flow to aggregate towards the bottom wall. In conclusion, Hartmann number might be regarded as a limiting parameter for heat flow.

### 5.4. Impact of the Geometrical Features

#### 5.4.1. Effect of Cylinder Placement

**Figure 8:**
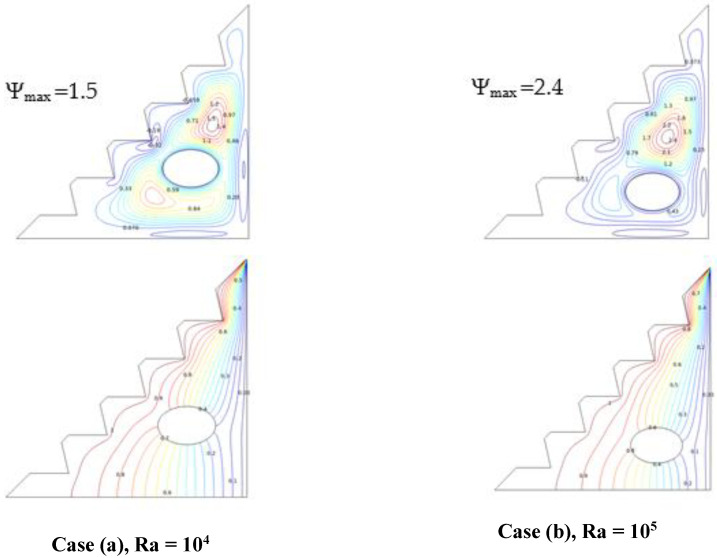
Streamlines and isotherms for Ha = 0, w = 0, and *ϕ* = 0.02.

Figure 8 highlights the distinctions between the two elliptical obstacle placements for 2% of the hybrid nano-fluid and no subjection to a magnetic field. It is observed that for Ra = $10^{4}$, the temperature distribution is directly affected by the location of the cylinder; the temperature variation in the configuration in the case of (b) provides better heat transfer, as it enables the flow to be distributed in the surrounding central section of the enclosure, with a large vortex that provides great stream-function values, which permits buoyancy forces to freely drive the hybrid nano-fluid flow, thereby significantly exploiting natural convection. In contrast to case (a), where the elliptical cylinder is positioned in the center of the enclosure, in this scenario, the flow is divided into two vortices: a large upper big vortex that offers a lower stream-function value than case (b) and a smaller one obstructed in the bottom of the enclosure, therefore obtaining less thermal transmission. According to these findings, it has been demonstrated that placing the obstacle at the bottom of the enclosure improves the heat-transfer rate.

#### 5.4.2. Effect of the Rotation of the Cylinder

**Figure 9:**
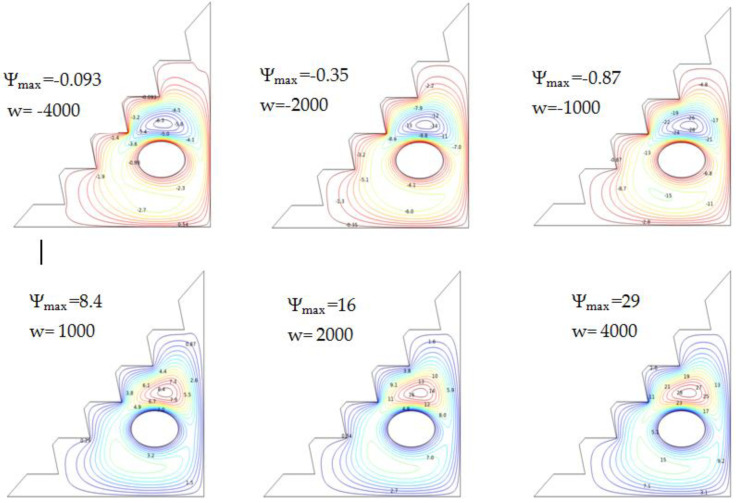
Streamlines via w and Ra = $10^{5}$, *ϕ* = 0.04, and Ha = 0.

Mixed convection was investigated at Ra=$10^{5}$, and for 4% of the hybrid nano-fluid, the findings are shown in Figure 9, which displays several streamlines, exhibiting the impact of the ellipse’s rotational velocity. A negative rotation value indicates a clockwise motion.

Leading the cylinder counter-clock wise reveals the benefits of the convective transfer; convection, due to the positive rotation, is concentrated around the cylinder, where this movement produces a increase in stream functions as an outcome of high values of velocity. As a result, large vortices are created; hence, the flow of the hybrid nano-fluid is accelerated. Natural convection is oriented towards the walls of the enclosure, where buoyancy forces are amplified. Therefore, augmenting the cylinder’s angular velocity in a counter-clockwise direction intensifies mixed convection and results in a higher heat-transfer rate. Clockwise movement, on the other hand, diminishes the heat-transfer rate, and fewer stream-function values are reported.

According to these findings, the direction of movement and the value of the angular rotational velocity of the cylinder may be employed as essential parameters for improving heat transmission.

#### 5.4.3. Effect of the Different Obstacles

**Figure 10:**
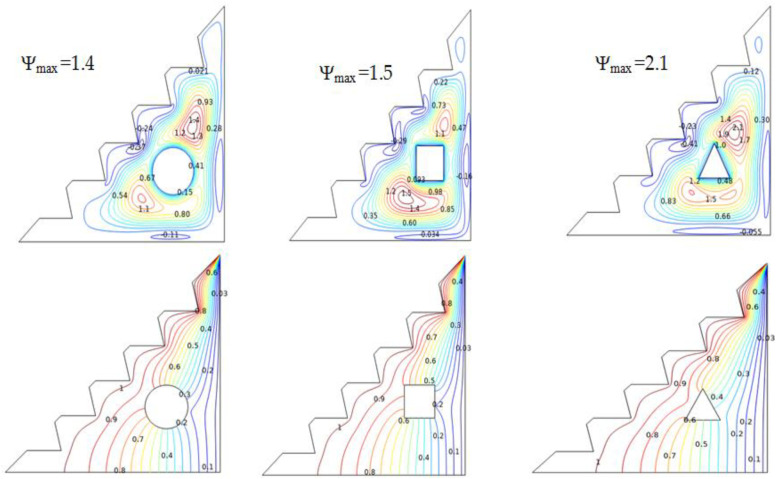
Streamlines and isotherms for w = 0, Ha = 0, Ra = 10^5^, and *ϕ* = 0.02.

The streamlines and isotherms produced and illustrated in Figure 10 investigate the influence of several obstacles in the considered geometry on the flow distribution. The findings demonstrate that the square and circular obstruction appear to slow the flow by producing small vortices compared to the triangular obstacle, which provides increased heat-transfer since it delivers the greatest values of stream function. The two vortices arestretched around this cylinder, which provides more space for flow distribution, thereby strengthening heat transfer. Temperature variations also significantly contribute to improved buoyant forces, which drive and boost natural convection.

**Figure 11:**
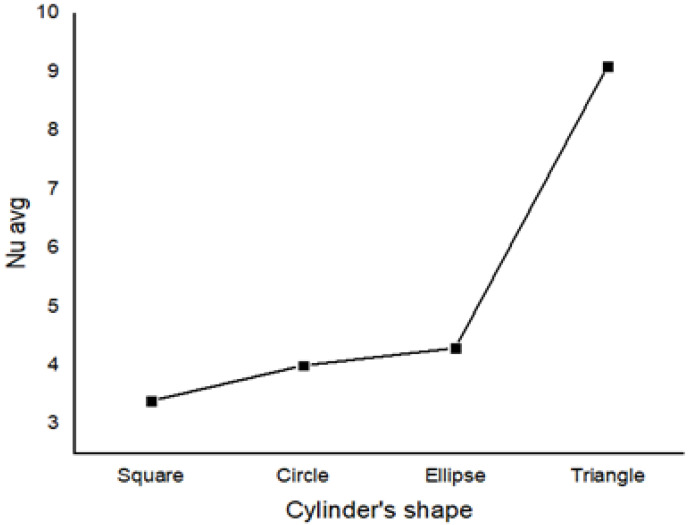
Average Nusselt values for w = 0, Ha = 0, Ra = 10^5^, and *ϕ* = 0.02.

Furthermore, Figure 11 demonstrates that the triangular barrier provides improved heat-transfer efficiency by presenting the highest peak of Nu_avg_ values when compared to the other cylinders. The geometrical features of the triangle and the uniform space provided around it make it easier for the hybrid nano-fluid to disperse, which can help amplify and alter the average Nu number and therefore convective transfer [68].

Additionally, the square, elliptic, and circular cylinders appear to have similar average Nusselt values; these cylinders should be further investigated by altering their radius.

## 6. Conclusions

In this paper, we performed a numerical study in order to investigate geometrical parameters and discussed which configuration is advantageous in terms of enhancement of convective heat transmission. The findings concerning streamlines, isotherms, and average Nusselt number obtained by altering Ra, *ϕ*, and Ha in the first examination documenting heat transfer in such a setup, using Cu- TiO_2_/ EG hybrid nano-fluid under magnetic-field influence reveal that incrementing the concentration of the working fluid from 0.02 to 0.08 improves the Nusselt number by 19%. Enhancing the Rayleigh number also accelerates the flow and strengthens the velocity field.

This enhancement is further boosted by the inclusion of counter-clockwise rotating cylinders in the enclosure, as well as triangular obstacles, which augment the Nu number by almost 120% compared to square, circular, and elliptical obstacles. However, this improvement is decremented when theenclosure is subjected to a magnetic field, as increasing Hartmann number reduced stream-function values and weakened the convective flow. Based on these findings, the following conclusions can be drawn:

- Rayleigh number and the volume fraction of the nanoparticles can be considered crucial features in modulating convection.
- The existence of a magnetic field, and therefore increasing Hartmann number, restricts heat transfer.
- Thermal transmission can be improved by using triangular obstacles.
- The angular velocity of the cylinder can alter the efficiency of the convective flow.
- The location of the obstacle is a key parameter to adjust the thermal transfer.

## Figures and Tables

**Table 1:** Magnetic flux-density range.

**Ha**	2550		100
**B_0_ (Tesla)**	1351	2702	5404

**Table 2:** Mesh-quality parameters.

Mesh	Extra Coarse	Coarse	Fine	Extra Fine
**Maximum element size (m)**	0.13	0.067	0.035	0.013
**Minimum element size (m)**	0.005	0.003	0.001	0.00015
**Curvature factor**	0.8	0.4	0.3	0.25
**Growth rate**	1.3	1.2	1.13	1.08
**Number of elements**	840	1984	3944	22184
**Average quality**	0.7110	0.7736	0.7803	0.8003

**Table 3:** Grid-independence test.

Mesh Quality	Nu	Nu Deviation %
0.7110	3.9	12.05%
0.7736	4.37	7.09%
0.7803	4.68	0.64%
0.8003	4.71	/

**Table 4:** Thermo-physical properties of Cu- TiO_2_/ EG hybrid nano-fluid.

	Cu	TiO_2_	EG
C_P_ (J. K^−1^·Kg^−1^)	385	686.2	2415
ρ(Kg·m^−3^)	8933	4250	1114
k(W. K^−1^·m^−1^)	401	8.95	0.252
β (K^−1^)	1.67 × $10^{-5}$	0.9 × $10^{-5}$	57 × $10^{-5}$
σ(Ohm·m)^−1^	5.96 × $10^{-7}$	2.38 × $10^{-6}$	5.5 × $10^{-6}$

## Data Availability

Data will be made available on request.

## Abbreviations

u, v	Velocity components (m·s^−1^)
U, V	Dimensionless velocity components
x, y	Cartesian coordinates (m)
X, Y	Dimensionless Cartesian coordinates
p	Pressure (N·m^−2^)
P	Dimensionless pressure
ρ	Density (Kg·m^−3^)
g	Gravitational acceleration (m·s^−2^)
T	Temperature (K)
T_avg_	Average temperature (K)
θ	Dimensionless temperature
α	Thermal diffusivity (m²·s^−1^)
υ	Kinematic viscosity (m²·s^−1^)
K	Permeability (H·m^−1^)
ε	Porosity
σ	Electric conductivity (Ohm m)^−1^
B_0_	Magnetic field density (Tesla)
k	Thermal conductivity ratio (W K^−1^ m^−1^)
Cp	Specific heat (J K^−1^ Kg^−1^)
β	Thermal expansion (K^−1^)
µ	Dynamic viscosity (Kg·m^−1^·s^−1^)
ϕ	Volume fraction of the nanoparticles
γ	Inclination angle of the magnetic field
w	Velocity of rotation(rad/s)
Ψ	Stream function
L	Length of the enclosure (m)
**Subscripts**	
h	Hot
c	Cold
EG	Ethylene glycol
Cu	Copper
TiO_2_	Titanium dioxide
MHD	Magneto-hydrodynamic
Nf	Nano-fluids
hnf	Hybrid nano-fluid
Bf	Base fluid
np	Nanoparticle
Max	Maximum
Fc	Forcheimer coefficient
Ra	Rayleigh
Nu	Nusselt
Ha	Hartmann
Da	Darcy
Pr	Prandtl
